# Arsenic Accumulation and Translocation in Mangrove (*Aegiceras corniculatum* L.) Grown in Arsenic Contaminated Soils

**DOI:** 10.3390/ijerph120707244

**Published:** 2015-06-26

**Authors:** Gui-Rong Wu, Hua-Long Hong, Chong-Ling Yan

**Affiliations:** 1 Key Laboratory of Ministry of Education for Coastal and Wetland Ecosystems, Xiamen University, Xiamen 361000, China; E-Mails: hzwgr510@163.com (G.R.W.); honghl.aries@gmail.com (H.L.H.); 2 College of Chemical and Biological Engineering, Hezhou University, Hezhou 542800, China

**Keywords:** arsenic, *Aegiceras corniculatum* L., accumulation, translocation, phytostabilization

## Abstract

Mangrove wetlands serve as both a sink and source for arsenic (As), as mangrove plants are able to uptake and accumulate As. The present study used pot experiments to evaluate As accumulation and translocation in mangrove (*Aegiceras* *corniculatum* L.) seedlings grown in As contaminated soils. Results indicated that *A. corniculatum* seedlings grew normally under As stress with minute growth inhibition and biomass reduction at different As treatment concentrations in a range of 0–150 mg·kg^−1^. As concentrations in roots, stems and leaves were increased with increasing As treatment concentrations, but As accumulated mainly in roots, with accumulation rates of 74.54%–89.26% of the total As accumulation. In particular, relatively high bioconcentration factor (BCF) in root (2.12–1.79), low BCF in stem (0.44–0.14) and leaf (0.06–0.01), and thereby a low translocation factor (TF) in stem/root (0.21–0.08) and leaf/root (0.02–0.008) were observed. These results demonstrated that *A. corniculatum* is an As excluder with the innate capacity to tolerate As stress and root tissues may be employed as a bio-indicator of As in polluted sediments. Additionally, *A. corniculatum* is a potential candidate mangrove species for As phytostabilization in tropical and subtropical estuarine wetlands.

## 1. Introduction

Arsenic (As) is a toxic environmental metalloid originating from geological and/or anthropogenic sources such as mining, burning of fossil fuels, use of fertilizers and agrochemicals, *etc.* [1,2]. As is a non-essential element for plants, also being highly phytotoxic and carcinogenic to humans through the food chain [3,4]. As is recognized as one of the most serious inorganic contaminants in natural water worldwide [5].

Mangroves are woody plants that grow on the coastal ecotone in tropical and subtropical latitudes, providing important ecological services including flood protection, prevention of shoreline erosion and salinity buffering [6,7]. Mangrove wetlands serve as both a sink and source for heavy metals (and also metalloids like As) in the coastline ecosystem, which prevents contaminant entry to adjacent waters but could increase the stress to mangrove plants [8,9]. High levels of As were found in mangrove sediments around the world, as 14.0 mg·kg^−1^ in Xiamen Bay (China) [10], 3.6–18.3 mg·kg^−1^ in the Sunderbans mangrove forest (India) [11], up to 70 mg·kg^−1^ in Espirito Santo (Brazil) [12], 0.52–35 mg·kg^−1^ in Sydney Estuary (Australia) [13]. Mangrove plants are able to survive under As stress, uptake and enrich As [14]. Recent field studies have shown that As is mainly accumulated in the roots and middle aerial part of *Avicennia marina* as compared to the upper part [15], As concentrations in fine nutritive roots of *Avicennia marina* were ~3-fold higher than those in sediments [13], while the uptake and bioaccumulation of As in *Kandelia obovata* are also significant [16]. However, field studies cannot provide more useful information on As accumulation and translocation in mangrove plants because of the frequent changes of As concentration and the lack of As concentration gradients in the natural environment. Thus, experimental study with more As concentration gradients, accessible for long-term observation and sampling, is urgently needed.

*Aegiceras corniculatum* L., an ecologically important tree species with a widespread distribution throughout the estuaries of southern China, was employed to evaluate As accumulation and translocation in mangrove plants. Data of plant growth and arsenic accumulation were collected through pot experiments with As addition to soils. The results of this study will provide useful information to improve our understanding of As tolerance in mangrove plants, and also its accumulation and translocation, and overall potential for As phytoremediation in tropical and subtropical estuarine wetlands.

## 2. Experimental Section

### 2.1. Soil Sampling

Surface soil samples (0–20 cm) were collected from mangrove sediments at Zhangjiang Estuary (23°55′ N, 117°25′ E, Fujian Province, China) for the pot experiments. Basic soil properties of the collected samples were analyzed based on standard methods [17], results are listed in Table 1.

**Table 1:** Properties of the soil used in the pot experiments. Values are mean ± SE (n = 5).

Organic Matter mg·kg^−1^	Available N mg·kg^−1^	Available P mg·kg^−1^	Available K mg·kg^−1^	Total As mg·kg^−1^	pH
3.17 ± 0.11	97.85 ± 4.67	28.41 ± 1.07	612.59 ± 18.54	14.32 ± 0.38	6.63 ± 0.08

### 2.2. Pot Experiments

Hypocotyls of *A. corniculatum* collected from the same location were pre-cultured in plastic pots (35 cm in diameter, 18.5 cm in-depth) filled with sea sand. The sea sand used was prewashed with concentrated HCl and rinsed thoroughly with tap water as described by Du *et al.* [18]. The pre-cultivation in a half-strength Hoagland’s nutrient solution lasted four weeks, and the solution was replaced once a week [19]. The experiment was conducted in a natural light greenhouse, and the salinity of irrigated water was maintained at 12.

After pre-cultivation, uniformly-sized seedlings were selected. Five seedlings were transplanted to each pot with ~5 kg either non-contaminated or As contaminated soil (as Na_2_HAsO_4_•7H_2_O). As concentrations were set based on the baseline concentration of As in the experiment soil (14.32 mg·kg^−1^). As treatment concentrations were increased about one-fold or two-fold the baseline As concentration, respectively, resulting in a total of seven treatments (0, 15, 30, 60, 90, 120 or 150 mg·kg^−1^ As). Each treatment had five replicates. The pot experiments were conducted for 180 days in natural light greenhouses, with a day/night temperature of 33/25 °C, and relative humidity of 65%/85%. Plants were watered to maintain soil moisture at 70%–80% of the field water holding capacity by addition of tap water during the experiments.

### 2.3. Sampling and Analysis

At harvest, seedlings were thoroughly washed with distilled water, and the shoot lengths, stem heights and leaf sizes were measured. Seedlings were then separated into root, stem and leaf tissues, and oven dried at 105 °C for 15 min, then at 70 °C until the samples reached constant weight. Oven-dried plant tissues were weighed, then ground to powder and passed through 100-mesh sieves.

Precisely 100 mg of plant material was placed into clean digestion tubes for digestion with 1 mL HNO_3_ and H_2_O_2_ (8:2, v/v) on a heating block at 180 °C for 1 h, and subsequently at 200 °C for 45–60 min so as to evaporate the samples to dryness. The residue was taken up in 10 mL demineralized water. Arsenic concentrations were measured by atomic absorption spectrophotometry (AAS: model AA-6800, Shimadzu, Kyoto, Japan), and calculated by dry weight (DW). Each sample was analyzed three times, and the mean value was calculated. The variances of duplicate measurements were less than 5%. Results obtained from these analyses were in good agreement with the certified values (±5%). The variances of samples in the same pot or in different pots were less than 5% and 10%, respectively.

The bioconcentration factor (BCF) reflects the ability of plants to accumulate arsenic, and is defined BCF = As_tissue_/As_soil_, where As_tissues_ = concentration of As in plant tissues (roots, stems and leaves) and As_soil_ = concentration of As in soil [16,20,21]. The translocation factor (TF) reflects the ability of plants to translocate As, and is defined TF = As_aerial_/As_root_, where As_aerial_ = concentration of As in plant’s aerial parts (stems and leaves) and As_root_ = Concentration of As in roots [22,23].

### 2.4. Data Analysis

Data are presented mean ± SE of five replicates (n = 5). Two-way analysis of variance (ANOVA) was done on all the data to confirm the variability of data and validity of results using SPSS software (19.0, SPSS, Inc., Chicago, IL, USA). Duncan’s multiple range test (DMRT) was performed to determine the significant difference between treatments at 0.05 probability level.

## 3. Results and Discussion

### 3.1. Plant Growth

The effects of As treatments on *A. corniculatum* seedling growth after 180 days are shown in Table 2. Treatments of 15–120 mg·kg^−1^ As had no significant impact on plant growth (*p* > 0.05), but at 150 mg·kg^−1^ As, root length decreased by 6.04%, stem height by 6.48% and life size by 18.16% as compared to the control. Plant biomass also decreased significantly at 150 mg·kg^−1^ As (*p* < 0.05), with root biomass decreasing by 11.15%, stem biomass by 11.46%, leaf biomass by 20.56%, resulting in the total biomass being decreased by 13.60% as compared to the control. The toxicity of large doses of heavy metals (e.g., Cu, Pb or Zn, or multiple heavy metals) on mangrove plants can cause toxic effects such as reduction in seedling height, leaf area, biomass and root growth, leaf chlorosis or necrosis, to finally induce mortality [24,25,26]. However, mangrove plants are considered to operate as excluder species for non-essential elements [20,27]. In the present study, *A. corniculatum* seedlings survived and grew normally at soil treatments of 15–120 mg·kg^−1^ As without those typical symptoms. These results demonstrated that *A. corniculatum* is an As excluder with the innate capacity to tolerate As stress.

**Table 2:** Effects of As treatments on *A. corniculatum* seedlings growth. Values are mean ± SE (n = 5). Different letters above same columns indicate significant differences at *p* < 0.05.

As Treatment mg·kg^−1^	Root Length cm	Stem Height cm	Leaf Size cm^2^	Root Biomass g DW	Stem Biomass g DW	Leaf Biomass g DW
0	17.05 ± 0.89 ^a^	14.67 ± 0.97 ^a^	12.39 ± 1.17 ^a^	2.03 ± 0.21 ^a^	3.09 ± 0.27 ^a^	1.67 ± 0.17 ^a^
15	16.98 ± 1.02 ^a^	14.50 ± 0.89 ^a^	11.97 ± 1.06 ^a^	2.05 ± 0.25 ^a^	3.13 ± 0.31 ^a^	1.68 ± 0.18 ^a^
30	17.10 ± 0.98 ^a^	14.70 ± 1.02 ^a^	11.74 ± 1.22 ^a^	2.08 ± 0.31 ^a^	3.14 ± 0.29 ^a^	1.71 ± 0.22 ^a^
60	17.07 ± 0.87 ^a^	14.76 ± 0.94 ^a^	12.09 ± 1.04 ^a^	2.07 ± 0.18 ^a^	3.12 ± 0.34 ^a^	1.68 ± 0.15 ^a^
90	16.96 ± 1.01 ^a^	14.59 ± 0.87 ^a^	11.95 ± 1.11 ^a^	2.04 ± 0.27 ^a^	3.10 ± 0.37 ^a^	1.64 ± 0.12 ^a^
120	16.85 ± 1.06 ^a^	14.43 ± 1.03 ^a^	11.84 ± 1.08 ^a^	1.97 ± 0.29 ^a^	3.06 ± 0.28 ^a^	1.61 ± 0.15 ^a^
150	16.02 ± 0.94 ^b^	13.72 ± 1.01 ^b^	10.14 ± 1.21 ^b^	1.81 ± 0.23 ^b^	2.73 ± 0.30 ^b^	1.32 ± 0.14 ^b^

### 3.2. As Accumulation

**Figure 1:**
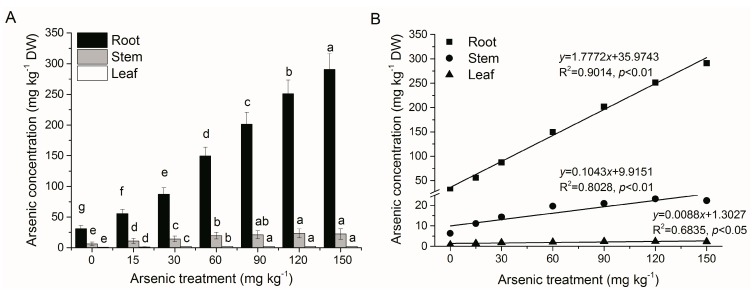
Effects of As treatments on (**A**) As concentrations in root, stem and leaf, and (**B**) linear relationship between As treatment concentrations and As concentrations in root, stem and leaf in *A. corniculatum* seedlings. Values are mean ± SE (n = 5). Different letters above comparable columns in Figure 1A indicate significant differences at *p* < 0.05.

**Figure 2:**
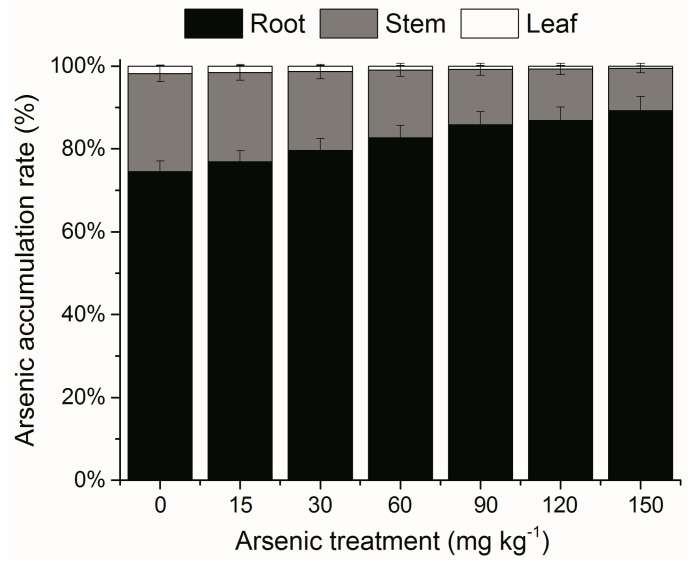
Effects of As treatments on As accumulation rates in root, stem and leaf in *A. corniculatum* seedlings. Values are mean ± SE (n = 5).

As accumulation in *A. corniculatum* differed with As treatment levels and plant tissues (Figure 1A). As concentrations in roots, stems and leaves were increased by increasing of As treatment concentrations (0–150 mg·kg^−1^), with significantly linear expression (R^2^ > 0.68, *p* < 0.05) (Figure 1B). As mainly accumulated in roots (30.29–294.85 mg·kg^−1^ DW), at levels which were 4.77–13.20-fold higher than those in stems, and 34.23–127.64-fold higher than those in leaves at soil As concentrations of 0–150 mg·kg^−1^. As accumulation rates in roots were 74.54%–89.26% of the total As accumulation (average 82.23% ± 2.06%), while those in stems were 23.68%–10.23% (average 16.68% ± 1.88%) and in leaves only 1.78%–0.51% (average 1.09% ± 0.18%) (Figure 2). An increasing trend of As accumulation rates in roots was found in roots with increasing As treatment concentrations, while opposite trends were found in stems and leaves. High As accumulation in roots was observed in other species like rice [28], ferns [29] and the mangrove species *Kandelia obovata* [16]. Transport of non-essential elements was interpreted by cell wall immobilization and/or sequestering of the epidermal layers [20]. Thus mangrove plants absorb and store non-essential elements in the perennial tissues, especially roots [30]. *A. corniculatum* root tissues might be a bio-indicator of As in polluted sediments, as As concentrations in roots are reflective of environmental levels.

### 3.3. Bioconcentration Factor and Translocation Factor

**Figure 3:**
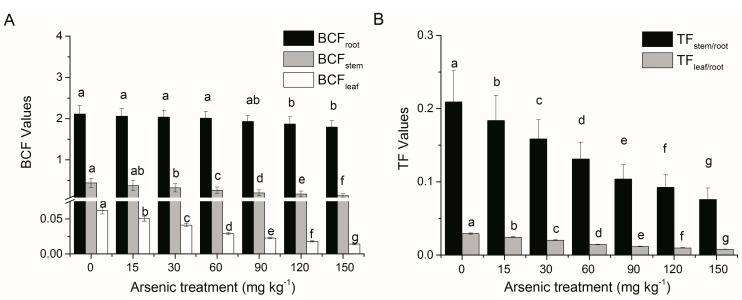
Effects of As treatments on (**A**) bioconcentration factor (BCF) of root, stem and leaf, and (**B**) translocation factor (TF) from root to stem and leaf in *A. corniculatum* seedlings. Values are mean ± SE (n = 5). Different letters above comparable columns indicate significant differences at *p* < 0.05.

**Figure 4:**
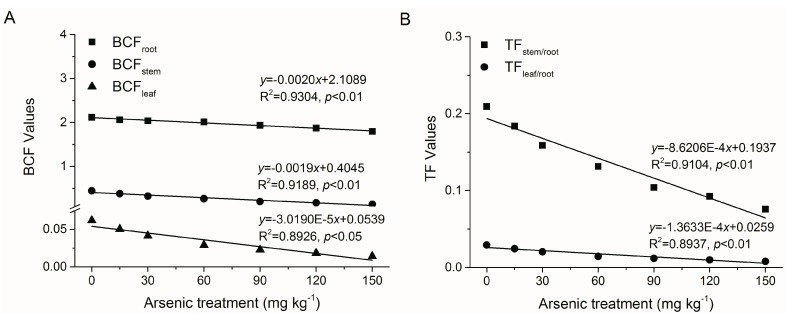
Linear relationship between As treatment concentrations and (**A**) bioconcentration factor (BCF) of root, stem and leaf, and (**B**) translocation factor (TF) from root to stem and root to leaf in *A. corniculatum* seedlings. Values are mean ± SE (n = 5).

BCF and TF were further employed to evaluate the ability of *A. corniculatum* to accumulate and translocate As. At treatments of 15–120 mg·kg^−1^ As, BCF_root_ was reasonable-high (ranged of 2.12–1.79), but BCF_stem_ (0.44–0.14) and BCF_leaf_ (0.06–0.01) were low, with values at 150 mg·kg^−1^ As decreased by 15.18%, 69.29% and 77.25% as compared to the control, respectively (Figure 3A). Meanwhile, TF_stem/root_ (0.21–0.08) and TF_leaf/root_ (0.02–0.008) were also extremely low, and values at 150 mg·kg^−1^ As decreased by 63.80% and 73.18% as compared to the control, respectively (Figure 3B). Significant negative correlations were found between TF or BCF values and soil As concentrations (R^2^> 0.89, *p* < 0.05) (Figure 4A,B). However, BCF_root_ values decreased slightly with increasing As treatment concentrations (0–150 mg·kg^−1^), while BCF_stem_ and BCF_leaf_ decreased sharply, resulting in great reductions in TF_stem/root_ and TF_leaf/root_ values. These results demonstrated that most of the As absorbed from the soil was retained in the roots and only small amounts were transported to the stems and leaves. Mangroves with high BCF_root_ (e.g., *Kandelia obovata* [16], *Avicennia marina* [13,31], *Phragmites australis* [32]) are appropriate candidates for phytostabilisation, retaining metallic inputs and thereby reducing transport to adjacent estuarine and marine systems. Besides, it was suggested that plant species with high BCF_root_ (>1) and low TFs (<1) could be considered as a potential candidate for the phytostabilization [33,34]. In the present study, high BCF_root_ (>1) and low TFs (<1) were observed at high soil As concentrations, suggesting that *A. corniculatum* is a potential candidate mangrove species for As phytostabilization in tropical and subtropical estuarine wetlands.

## 4. Conclusions

In the present study, *A. corniculatum* seedlings survived and grew normally without typical symptoms of As toxicity, demonstrating that *A. corniculatum* is an As excluder with an innate capacity to tolerate As stress. As was mainly accumulated in roots, with As concentrations increasing with increasing As treatment concentration, indicating that *A. corniculatum* root tissues may be employed as a bio-indicator of As in polluted sediments. Additionally, relatively high BCF_root_ (>1) and low TFs (<1) suggested that *A. corniculatum* is a potential candidate mangrove species for As phyto-stabilization in tropical and subtropical estuarine wetlands.
